# Heat shock protein family D member 1 in boar spermatozoa is strongly related to the litter size of inseminated sows

**DOI:** 10.1186/s40104-022-00689-0

**Published:** 2022-04-15

**Authors:** Won-Ki Pang, Ji-Hyun Son, Do-Yeal Ryu, Md Saidur Rahman, Yoo-Jin Park, Myung-Geol Pang

**Affiliations:** Department of Animal Science & Technology and BET Research Institute, Chung-Ang University, Anseong, Gyeonggi-do 17546 Republic of Korea

**Keywords:** Fertilisation, HSPD1, Male fertility, Sperm motility, Sperm RNA

## Abstract

**Background:**

Sperm quality evaluation is the logical first step in increasing field fertility. Spermatozoa contain cytoplasmic organelles and biomolecules known as sperm-intrinsic factors, which play key roles in sperm maturation, sperm-oocyte fusion, and embryo development. In particular, sperm membrane proteins [e.g., arginine vasopressin receptor 2, beta-actin, prohibitin, and heat shock protein family D member 1 (HSPD1)] and RNA could be used as functional indicators of male fertility. We sought to clarify the effects of differential mRNA expression of selected genes on several fertilisation parameters, including sperm motility, motion kinematics, capacitation, and litter size, in a porcine model.

**Results:**

Our results demonstrated that *HSPD1* expression was significantly correlated with male fertility, as measured by the litter size of inseminated sows. The expression of *HSPD1* mRNA was linked to sperm motility and other motion kinematic characteristics. Furthermore, *HSPD1* had a 66.7% overall accuracy in detecting male fertility, and the high-litter size group which was selected with the *HSPD1* marker had a 1.34 greater litter size than the low-litter size group.

**Conclusions:**

Our findings indicate that *HSPD1* might be a helpful biomarker for superior boar selection for artificial insemination, which could boost field fertility.

**Supplementary Information:**

The online version contains supplementary material available at 10.1186/s40104-022-00689-0.

## Background

Artificial insemination (AI) has been applied globally to breed various types of livestock. More than 80% of cattle and swine production worldwide depends on AI [1]. Therefore, AI failure can cause considerable economic damage to the animal industry. Moreover, approximately 50% of all infertility cases in humans and animals are caused by male factors [1], and only 50% of inseminations result in successful full-term pregnancies [2]. Thus, male fertility is a critical factor to consider when assessing AI failure in the livestock industry. To increase the success rate of AI, new technologies must be developed to optimise sperm quality.

Spermatozoa carry not only the paternal genome, but also several intrinsic factors that modulate early development after fertilisation, including cytoplasmic organelles and biomolecules, such as proteins and RNAs [3–5]. These sperm-intrinsic factors (SIFs) are involved in critical steps of development, such as sperm maturation, sperm-oocyte fusion, and embryo development [4, 6]. Proteins and RNAs in spermatozoa directly affect pre- and post-fertilisation processes. For instance, proteins secreted from the intraluminal compartment of the epididymis interact with sperm surface proteins and induce sperm maturation [7]. Moreover, several sperm cell functions and developmental milestones, such as capacitation, acrosome reaction, sperm penetration, and sperm-oocyte fusion, are strictly controlled by sperm proteins [8]. Several studies have suggested that sperm proteins and RNAs play important roles in fertilisation and pre-implantation embryonic development [8–11]. Several transcriptomic studies of spermatozoa have shown that differential expression of RNA is related to diverse semen traits [12–16]. Although numerous RNAs have been associated with male fertility, further research is needed before changes can be implemented in the field.

Several compelling studies have successfully demonstrated the applicability of sperm proteins and RNA as biomarkers of male fertility [2, 17–20]. The vast majority of sperm membrane proteins have been identified as biomarkers of male fertility outcomes in these studies. Sperm membrane proteins play crucial roles in developmental biology, including protein receptor signalling, flagella movement control, ion homeostasis regulation, and sperm-zona pellucida interactions [20]. In particular, differential expression of arginine vasopressin receptor 2 (AVPR2), beta-actin (ACTB), prohibitin (PHB), and heat shock protein family D member 1 (HSPD1) proteins, all of which are classified by the Gene Ontology database as integral membrane components, has been linked to male fertility outcomes [17]. AVPR2, ACTB, and PHB are closely related to sperm motility and affect male fertility. Arginine vasopressin affects the male reproductive tract, sperm count, and motility in mice [21]. AVPR2 is the G-protein-coupled receptor of arginine vasopressin, and *AVPR2* mRNA is found in the vas deferens epithelium of humans and pigs [22] and the tail mid-piece and the acrosome region of mouse spermatozoa [21]. ACTB is a major component of the cytoskeleton and is involved in many crucial cellular processes [23]. Differential expression of the ACTB protein has been linked to male fertility outcomes in both human and porcine spermatozoa [17, 24, 25]. PHB is a sperm mitochondrial protein that modulates mitochondrial structure and functions as a molecular chaperone [26]. A recent study demonstrated that PHB interacts with protein kinase B in the mitochondrial sheath of murine spermatozoa and controls motility by activating the phosphoinositide 3-kinase/serine-threonine kinase (PI3K/AKT) signalling pathway [27]. HSPD1 is a major target for capacitation-associated tyrosine phosphorylation, which exposes the zona pellucida receptor to the cell surface of spermatozoa [28, 29].

Although AVPR2, ACTB, PHB, and HSPD1 are known to be linked to male fertility, the effects of their corresponding mRNA levels on fertility are not well understood. Considering the crucial role of proteins in male fertility, we hypothesised that the mRNAs encoding these proteins may play a key role in pre- and post-fertilisation processes. To test this hypothesis, we used boar sperm samples. Pigs offer unique advantages over other well-established species for developmental biology studies because pigs share major characteristics with humans, despite being a polytocous (i.e., multiparous) species. Thus, this polytocous trait provides more comprehensive and convincing information on male fertility after AI in a large number of sows. To investigate the physiological role of sperm mRNA, we focused on elucidating the direct relationship between the mRNA expression of selected genes in pre-fertilisation parameters (sperm motility, motion kinematics, and capacitation status) and in vivo fertility.

## Methods

All procedures involving animals were approved by the Institutional Animal Care and Use Committee of Chung-Ang University (Approval No. 2017–00018) and performed in accordance with the corresponding guidelines. All methods were performed according to relevant guidelines and regulations.

### Experimental design

All semen samples were obtained and processed as described below. Semen samples (*n =* 27) were acquired from a farm (Sunjin Co., Danyang, Korea).

First, to compare the parameters in representative groups, samples from high- (average litter size 13.15 ± 0.39, *n =* 3) and low- (average litter size 11.50 ± 0.10, *n =* 3) litter sizes were selected based on the average litter size (total piglets/total breeding). Pre-fertilisation parameters (motility, motion kinematics, and capacitation status) and mRNA expressions of *AVPR2, ACTB, PHB*, and *HSPD1* were assessed in the high- and low-litter size groups.

Subsequently, randomly selected boar sperm samples (*n =* 21) were examined to elucidate the correlation between mRNA expression levels and pre-fertilisation parameters (sperm motility, motion kinematics, and capacitation status) and in vivo fertility.

### Boar sperm preparation

Upon collection, the sperm samples were immediately transferred to a constant temperature container (17 °C) and stored until the downstream processing steps [30]. All semen samples were centrifuged at 500×*g* for 20 min with a discontinuous 70% (v/v) and 35% (v/v) Percoll gradient (Sigma-Aldrich, St Louis, MO, USA) to remove seminal plasma as well as immotile and dead spermatozoa [31]. The isolated live spermatozoa were then incubated at 37 °C in 5% CO_2_ modified tissue culture medium 199 (mTCM 199; 0.91 mmol/L sodium pyruvate, 3.05 mmol/L d-glucose, 2.92 mmol/L calcium lactate, and 2.2 g/L sodium bicarbonate; Sigma-Aldrich) for 30 min [30, 32].

### Computer-assisted sperm analysis (CASA)

Boar sperm motility (%) and motion kinematics were analysed using a CASA system (SAIS-PLUS VERSION 10.1; Medical Supply, Seoul, Korea) [33]. After incubation, 10 μL of the sperm sample was placed in a Makler counting chamber (Sefi Medical Instruments, Haifa, Israel), which was then placed on a preheated heat block at 37 °C [32, 34, 35]. Sperm motility, hyperactivated motility (HYP), curvilinear velocity (VCL), straight-line velocity (VSL), average path velocity (VAP), mean amplitude of lateral head displacement (ALH), beat cross frequency (BCF), linearity (LIN), and wobble (WOB) were determined [32]. Each sample was observed using phase-contrast microscopy with a 10× objective lens [36].

### Combined Hoechst 33258/chlortetracycline fluorescence assessment of capacitation status

The capacitation status of boar spermatozoa was examined with a dual staining method using a combined Hoechst 33258/chlortetracycline fluorescence staining process [30, 37]. The samples were first incubated in mTCM 199 and centrifuged at 400×*g* for 10 min at room temperature. The supernatant was discarded, and then 135 μL of phosphate-buffered saline (PBS) and 15 μL of H33258 solution were added. The samples were gently mixed and incubated for 10 min at room temperature. Excess dye was inactivated with 250 μL of 2% (w/v) polyvinylpyrrolidone (Sigma-Aldrich) in PBS. After centrifugation at 400×*g* for 10 min, the supernatant was discarded, and then the pellet was resuspended in 600 μL of PBS and 600 μL of chlortetracycline (CTC) fluorescence solution (750 mmol/L CTC in 5 μL buffer; 20 mmol/L Tris, 130 mmol/L sodium chloride (NaCl), and 5 mmol/L cysteine, pH 7.4; Sigma-Aldrich) [38]. The stained samples were counted using a Microphot-FXA microscope (Nikon, Tokyo, Japan) under epifluorescence illumination using ultraviolet BP 340–380/LP 425 and BP 450–490/LP 515 excitation/emission filters for H33258 and CTC, respectively. Capacitation status was quantified on approximately 400 spermatozoa per slide for each sample. Capacitation status was further classified into four categories: live non-capacitated (F; green fluorescence distributed evenly throughout the sperm head), live capacitated (B; green fluorescence over the acrosome region and a dark post-acrosome region), acrosome-reacted (AR; showing no fluorescence over the head), and dead (D; nuclei with blue fluorescence within the sperm head) [37].

### RNA extraction, cDNA synthesis, and reverse transcription-quantitative polymerase chain reaction (RT-qPCR)

RNA extraction, cDNA synthesis, and RT-qPCR were conducted as described previously [39]. Briefly, all samples were washed with PBS, centrifuged at 10,000×*g* for 10 min, and stored at − 80 °C prior to RNA extraction. Each sperm sample was counted, and then sperm concentrations were adjusted to 50 × 10^6^ cells/mL with fresh PBS. The samples were then centrifuged at 13,000×*g* for 10 min at 4 °C, and the supernatant was then removed. Sperm pellets were lysed using a lysis buffer (PureLink™ RNA Mini Kit; Invitrogen, Carlsbad, CA, USA) containing 40 μL/mL β-mercaptoethanol (Sigma-Aldrich) and then homogenised with 20 G needles. After mixing the homogenised mixture for 2 min, 500 μL of TRIzol reagent (Invitrogen) was added to the sperm sample. The sample was then kept at room temperature for 5 min, and then 200 μL of chloroform (Sigma-Aldrich) was added, and the samples were mixed vigorously by hand for 20 s. The samples were incubated at room temperature for another 5 min and then centrifuged at 12,000×*g* for 25 min at 4 °C. Next, 500 μL of the upper phase (which contained RNA) was carefully transferred to a fresh 1- mL tube, and an equal amount of 100% pure ethanol was then added. The mixture was then mixed by pipetting and processed according to the manufacturer’s instructions. RNA was eluted in 20 μL nuclease-free water. RNA concentrations and 260/280 ratios were measured using an Epoch microplate spectrophotometer (BioTek, Winooski, VT, USA). cDNA synthesis was performed using the PrimeScript 1^st^ strand cDNA Synthesis Kit (Takara Bio, Inc., Shiga, Japan) following the manufacturer’s instructions. RT-qPCR was performed using the *AVPR2*-, *ACTB*-, *PHB*-, *HSPD1*-, glyceraldehyde 3-phosphate dehydrogenase (*GAPDH*)-, and peptidylprolyl isomerase A (*PPIA*)-specific primers, which were designed for *Sus scrofa* (Additional file 1: Table S1). *GAPDH* and *PPIA* were used as reference genes [40], and RT-qPCR data were analysed using the delta-delta Cq method [41].

$$ \Delta  \mathrm{Cq}=\mathrm{Cq}\left(\mathrm{a}\ \mathrm{target}\ \mathrm{gene}\right)-\mathrm{Cq}\left(\mathrm{a}\ \mathrm{reference}\ \mathrm{gene}\right) $$

$$ \mathrm{Relative}\ \mathrm{expression}\ \mathrm{of}\ \mathrm{target}\ \mathrm{gene}={2}^{-\left(\Delta  Cq\left(a\  target\ sample\right)-\Delta  Cq\left(a\  reference\ sample\right)\right)} $$

The standardised annealing temperature of the designed primers was 60 °C. A standard curve analysis was performed for each gene to determine the PCR efficiency. Melt curve analysis was conducted to examine the single amplification, and the size of the PCR products was determined using gel electrophoresis [39].

### Western blotting

Protein quantification of HSPD1 was performed by Western blotting [21]. Briefly, 100 × 10^6^ sperm cells were lysed with a buffer containing 5% 2-mercaptoethanol (Sigma-Aldrich). The lysate was centrifuged at 10,000×*g* for 10 min at room temperature. The supernatant was boiled and stored for electrophoresis. Proteins were separated using sodium dodecyl sulphate-polyacrylamide gel electrophoresis (SDS-PAGE). Separated proteins were transferred onto polyvinylidene fluoride membranes (Amersham, Piscataway, NJ, USA). For protein detection, anti-HSPD1 antibody (Abcam, Cambridge, UK) was used at a 1:20,000 ratio. Horseradish peroxidase (HRP)-conjugated rabbit IgG (1:3000; Cell Signaling Technology, Danvers, MA, USA) was used to detect HSPD1 level. Protein α-tubulin was quantified as an internal control with anti-α-tubulin mouse antibody (1:10,000; Abcam) and HRP-conjugated mouse IgG (1:3000; Cell Signaling Technology). The HRP signal was visualised on an X-ray film using chemiluminescence. The X-ray film was scanned using a GS-800-calibrated imaging densitometer (Bio-Rad, Hercules, CA, USA) and analysed using Quantity One software (Bio-Rad). The ratio of HSPD1/α-tubulin levels was calculated as the relative expression level of each sample.

### Measurement of male fertility

AI was conducted to measure the male fertility outcomes. Environmental conditions were maintained at 20 ± 5 °C with ventilation and a 16 h light and 8 h dark photoperiod. The average number of AI per boar was 22.95 ± 1.24 (18–38). Semen samples from boars were diluted with Beltsville thawing solution (100 mL per 30 × 10^6^ sperm cells) and stored at 17 °C until required for insemination.

### Quality assessment of genes as the indicators of male fertility

Sensitivity, specificity, negative predictive value (NPV), and positive predictive value (PPV) were evaluated using screening tests [37, 42, 43]. Sensitivity was defined as the ratio of boars showing true-positive results (i.e., the percentage of boars for which we could accurately identify the litter sizes). In contrast, specificity was defined as the percentage of boars that exhibited true-negative results. PPV and NPV were defined as the rate of boars exhibiting positive or negative results, respectively, when the litter size was ≥ 12.68 or < 12.68 (average litter size of samples).

### Statistical analysis

All data were analysed using SPSS v1.8 (SPSS Inc., Chicago, IL, USA), and all parameters were confirmed for normality using the Shapiro–Wilk test. All comparisons between two groups were analysed using Student’s two-tailed *t*-tests and the homogeneity of variance test (Levene’s test). Correlations were identified using Pearson’s correlation coefficients for the groups that exhibited a normal distribution (*P* ≥ 0.05). In groups that failed the normality tests (*P* < 0.05), the Spearman correlation coefficient was used [44]. The prognostic power of fertility parameters as a function of *HSPD1* mRNA expression was evaluated using the receiver operating characteristic (ROC) curve, and optimal cut-off values were generated based on the highest sensitivity and specificity values determined from ROC analysis [45, 46]. The accuracy of *HSPD1* in assessing male fertility outcomes was determined based on sensitivity and specificity. All numerical data are reported as the mean ± standard error of the mean (SEM), and *P* < 0.05 were considered statistically significant.

## Results

### Pre-fertilisation parameters (motility, motion kinematics, and capacitation status) and gene expression in the high- and low-litter size boar groups

**Fig. 1:**
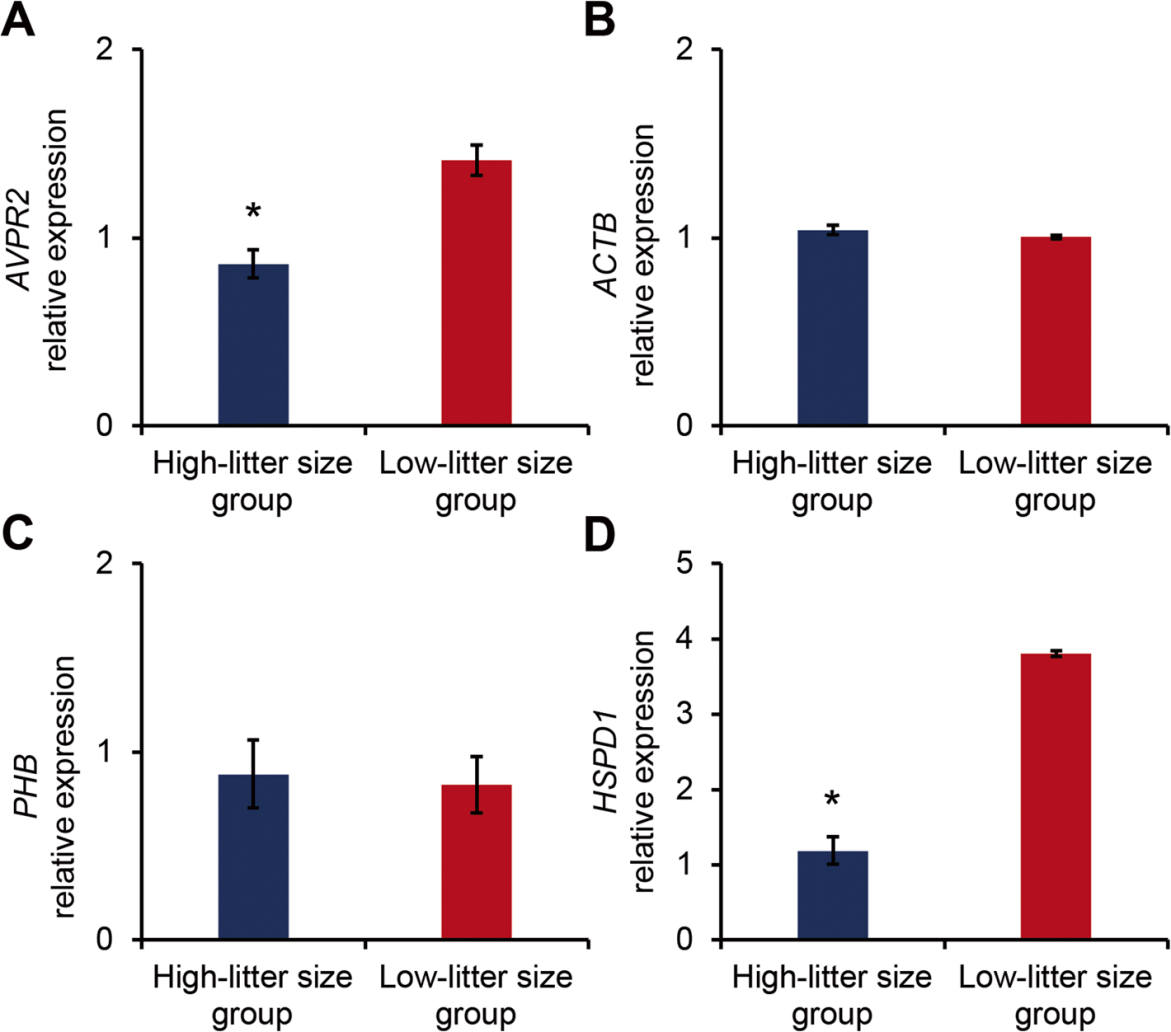
Beta-actin (*ACTB*), prohibitin (*PHB*), heat shock protein family D member 1 (*HSPD1*), and arginine vasopressin receptor 2 (*AVPR2*) mRNA expression in high- and low-litter size boar spermatozoa. Differences in marker candidate gene expression in the high-litter size (*n =* 3) and low-litter size (*n =* 3) spermatozoa groups based on the average litter sizes. **A*** AVPR2* mRNA expression in boar spermatozoa with high- and low-litter sizes. **B*** ACTB* mRNA expression in boar spermatozoa with high- and low-litter sizes. **C*** PHB* mRNA expression in boar spermatozoa with high- and low-litter sizes. **D*** HSPD1* mRNA expression in boar spermatozoa with high- and low-litter sizes. Relative expression was normalised to *GAPDH* expression. The data are expressed as the mean ± standard error of the mean (SEM); ^*^P < 0.05

CASA and H33285/CTC dual staining were conducted to assess sperm motility, motion kinematics, and capacitation status as the pre-fertilisation potential of boar spermatozoa. As summarised in Table 1, no significant differences in sperm motility, motion kinematics, and capacitation status were observed between the high- and low-litter size boar groups (*P* < 0.05). The PCR efficiency of the designed primers ranged from 89% to 107% (Additional file 1: Fig. S1). PCR amplicon size after reaction with the designed primers matched the predicted size, and all melt curve analyses showed a single peak (Additional file 1: Fig. S2). Among the selected genes, *AVPR2* and *HSPD1* mRNA expression levels were significantly different between sperm samples (Fig. 1 and Additional file 1: Fig. S3). Specifically, *AVPR2* and *HSPD1* mRNA expression levels were higher in the low-litter size boar group (*P* < 0.05; Fig. 1A and D and Additional file 1: Fig. S3A and D).

**Table 1:** Male fertility parameters of high- and low-litter size spermatozoa

Parameter		High-litter size group	Low-litter size group
Litter size		13.15 ± 0.39*	11.50 ± 0.10
Motility and motion kinematics	MOT, %	84.13 ± 2.21	90.7 ± 0.68
	HYP, %	13.28 ± 0.55	15.66 ± 2.15
	VCL, μm/s	141.05 ± 10.69	149.68 ± 4.73
	VSL, μm/s	66.29 ± 17.43	71.02 ± 1.01
	VAP, μm/s	76.33 ± 13.51	83.23 ± 1.39
	LIN, %	45.73 ± 8.27	47.49 ± 0.91
	BCF, Hz	11.93 ± 0.44	11.49 ± 0.27
	WOB, %	53.34 ± 5.18	55.67 ± 0.27
	ALH, μm/s	6.37 ± 0.62	6.64 ± 0.13
Capacitation status	AR, %	0.22 ± 0.22	1.28 ± 0.51
	F, %	91.48 ± 1.02	91.51 ± 3.4
	B, %	8.28 ± 1.07	7.2 ± 3.39

*MOT* motility, *HYP* hyperactivated motility, *VCL* curvilinear velocity, *VSL* straight-line velocity, *VAP* average path velocity, *BCF* beat cross frequency, *LIN* linearity, *WOB* wobble, *ALH* amplitude of lateral head displacement. AR, acrosome-reacted spermatozoa; F, non-capacitated spermatozoa; B, capacitated spermatozoa; *P < 0.05

### Correlation analysis between gene expression and pre-fertilisation parameters and litter size

**Fig. 2:**
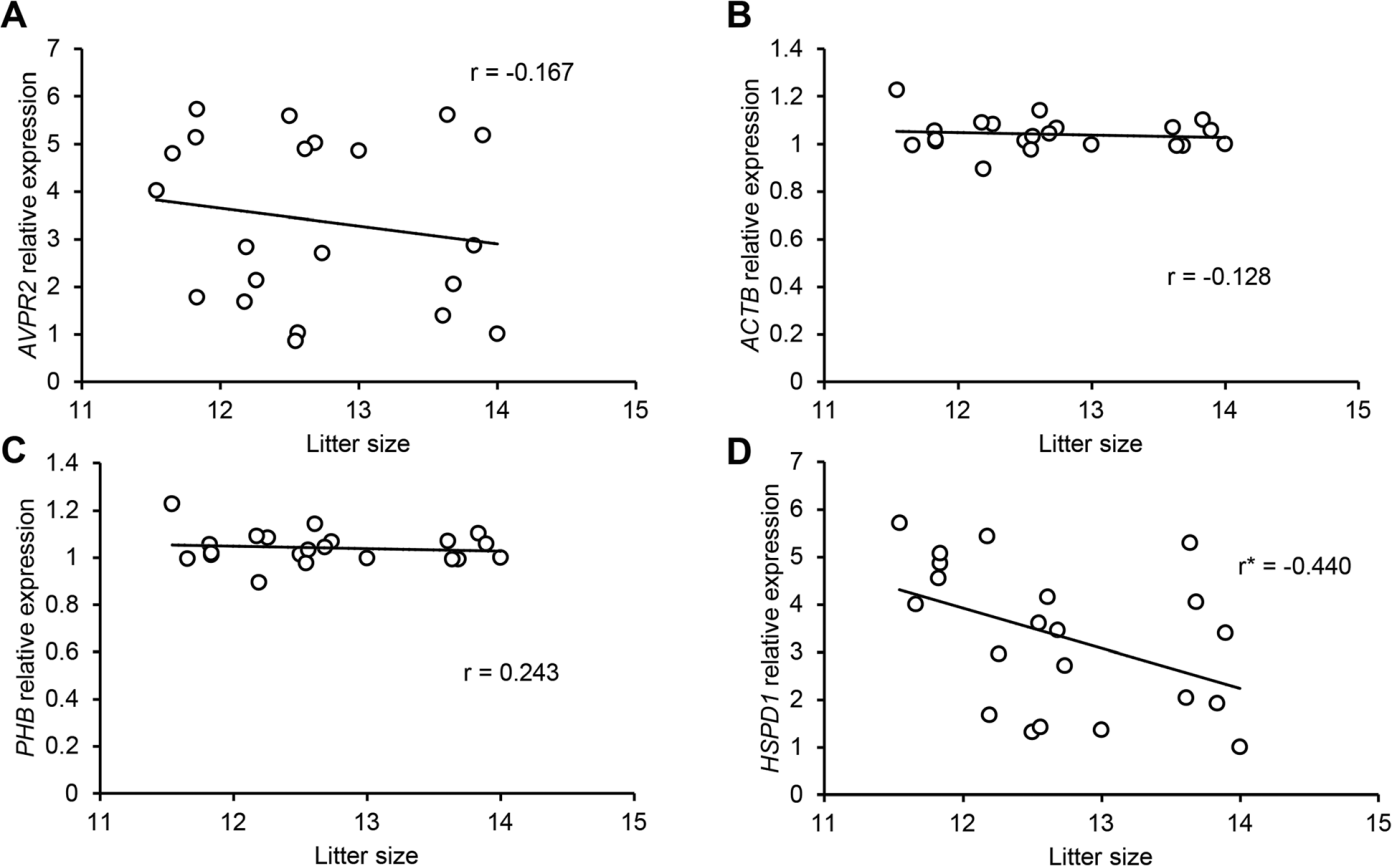
Correlation analysis between gene expression and pre-fertilisation parameters. **A** Linear regression of *AVPR2* mRNA expression and litter size. **B** Linear regression of *ACTB* mRNA expression and litter size. **C** Linear regression of *PHB* mRNA expression and litter size. **D** Linear regression of *HSPD1* mRNA expression and litter size. r, Pearson correlation coefficient; ^*^P < 0.05, calculated via the linear regression test

The results for the normality test of every parameter (pre-fertilisation parameters, litter size, and gene expression) from 21 boars are provided in Additional file 1: Table S2. A correlation analysis was conducted to identify the interactions between all parameters in 21 randomly selected boars (Figs. 2 and 4A–D and K). The mRNA expression of *HSPD1* was significantly correlated with litter size (*R* = − 0.440; Fig. 2D and Additional file 1: Table S3). Moreover, *HSPD1* expression was positively correlated with sperm motility (%) and several motion kinematics, including VCL (μm/s), VAP (μm/s), and ALH (μm/s) (Fig. 4A–D and Additional file 1: Table S3).

**Fig. 3:**
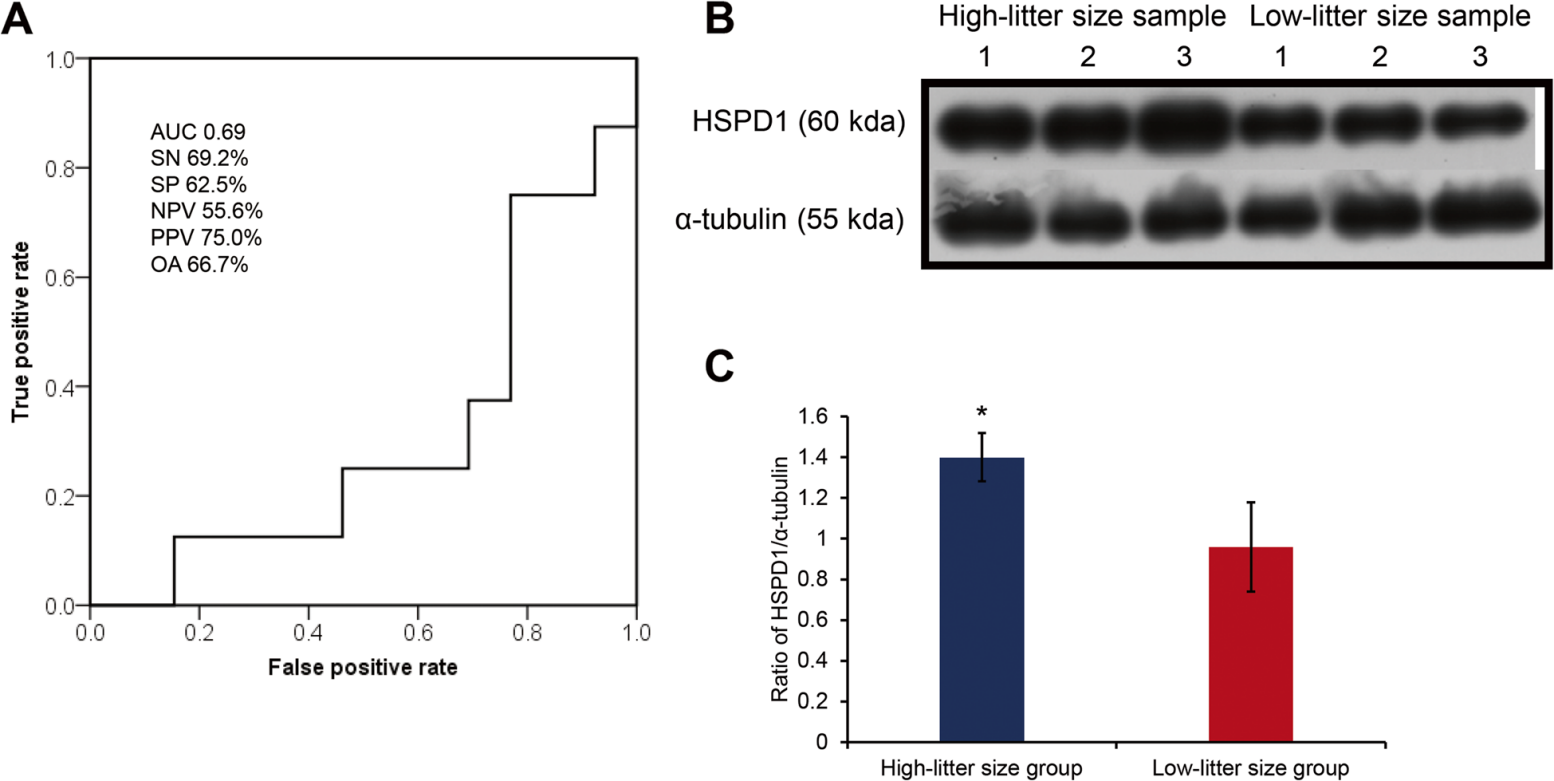
Quality assessment of *HSPD1* mRNA marker and protein expression. **A** Receiver operating characteristic (ROC) curve of *HSPD1* mRNA expression versus litter size. All the predictive parameters were calculated based on the average litter size (12.68) of samples. AUC, Area under the curve. Sensitivity (SN) is the percentage of boars showing true-positive results when tested with mRNA expression. Specificity (SP) is the percentage of boars showing true-negative results. The positive predictive value (PPV) is the percentage of boars that tested positive and also exhibited a true-positive litter size. The negative predictive value (NPV) is the percentage of boars that tested negative or simultaneously had a true-negative litter size. OA, Overall accuracy. **B** Western blotting image of HSPD1 and α-tubulin proteins. **C** Relative expression of HSPD1 in high- and low-litter size groups. The data are expressed as the mean ± SEM; ^*^P < 0.05

**Fig. 4:**
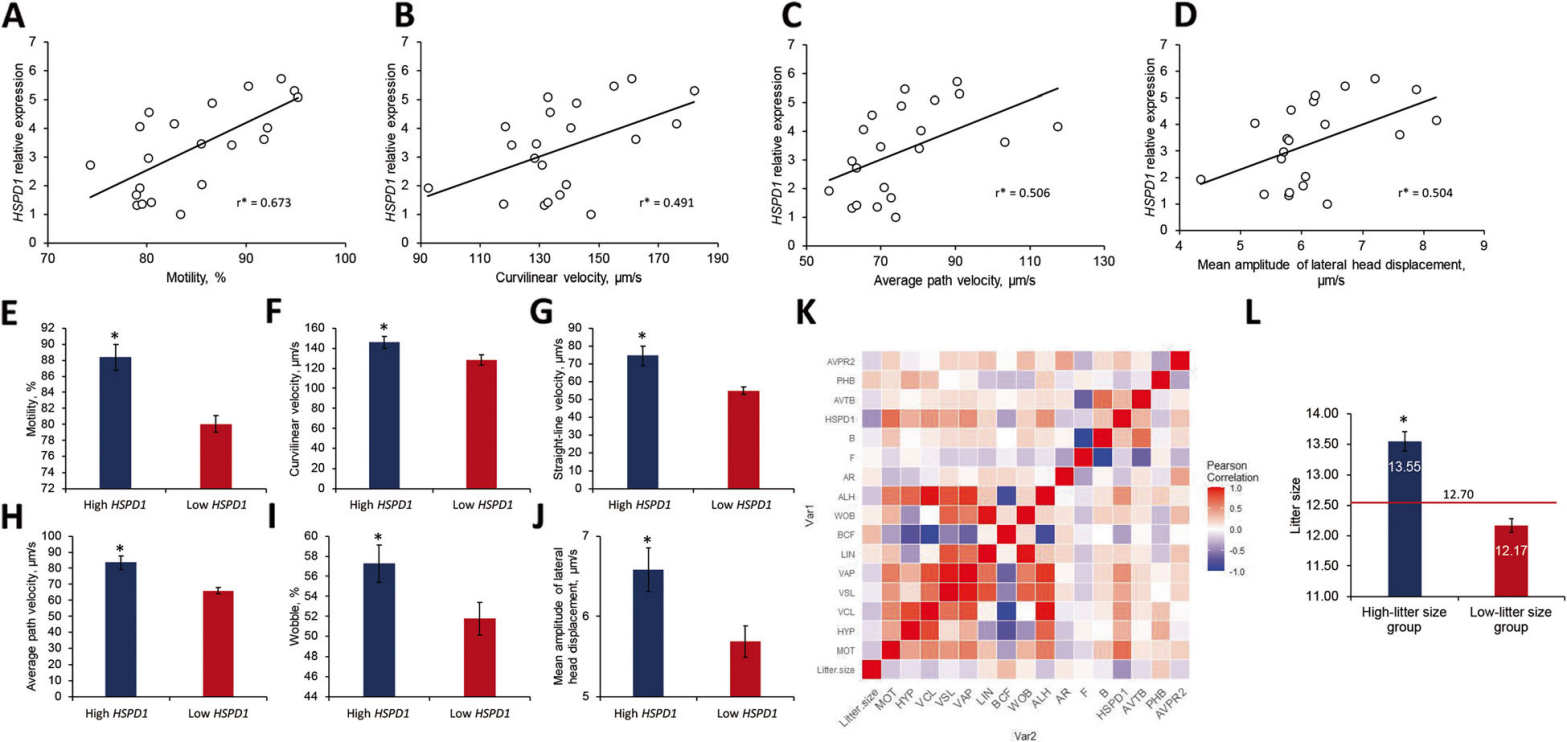
Fertility parameters linked to gene expression dysregulation. **A** Linear regression of *HSPD1* expression and motility (%). **B** Linear regression of *HSPD1* expression and curvilinear velocity (μm/s). **C** Linear regression of *HSPD1* expression and average path velocity (μm/s). **D** Linear regression of *HSPD1* expression and mean amplitude of lateral head displacement (μm/s). **E** Difference of motility in high- and low-*HSPD1* expression groups. **F** Difference of curvilinear velocity (μm/s) in high- and low-*HSPD1* expression groups. **G** Difference of straight-line velocity (μm/s) in high- and low-*HSPD1* expression groups. **H** Difference of average path velocity (μm/s) in high- and low-*HSPD1* expression groups. **I** Difference of wobble (%) in high- and low-*HSPD1* expression groups. **J** Difference of mean amplitude of lateral head displacement (μm/s) in high- and low-*HSPD1* expression groups. The average values of each fertility parameter were compared based on the cut-off values of *HSPD1* expression (3.1798) from the ROC curves. **K** Correlation heatmap of all parameters. **L** Average litter size of high- and low-litter size groups separated by *HSPD1* mRNA expression. The data are expressed as the mean ± SEM; ^*^P < 0.05

### Quality of genes as the indicators of male fertility

*HSPD1* was characterised for quality assurance. The sensitivity, specificity, NPV, PPV, and overall litter size prediction accuracy of *HSPD1* were 69.2%, 62.5%, 55.6%, 75.0%, and 66.7%, respectively (Fig. 3A). The area under the curve (AUC) for the *HSPD1* ROC curve was 0.69 (Fig. 3A). The samples were divided into high- and low-*HSPD1* expression groups based on the cut-off value of the ROC curve. In the *HSPD1* high-expression group, the motility (%), VCL (μm/s), VSL (μm/s), VAP (μm/s), WOB (%), and ALH (μm/s) were significantly different (*P* < 0.05) from the those of the *HSPD1* low-expression group (Fig. 4E–J and Additional file 1: Table S4). According to the cut-off value of *HSPD1* expression, the litter size difference between the high- and low-litter size groups was 1.34 (*P* < 0.05; Fig. 4L). HSPD1 protein was highly expressed in the low-litter size group (*P* < 0.05; Fig. 3B and C).

## Discussion

A substantial proportion of the livestock industry relies on AI-based breeding, and the fertilisation success rate can be affected by sperm quality. Therefore, sperm quality assessment is critical for predicting male fertility. To accurately investigate male fertility markers to increase field fertility, a porcine model was selected for the current study. The importance of the porcine model for biomedical research has been widely acknowledged [47]. Pigs are also uniquely well-suited for developmental studies because of the similarity between porcine and human genomes and the availability of numerically well-organised AI fertility data [48, 49]. The AI of pigs has rendered a plethora of valuable information on male fertility. Therefore, the porcine model provides unique advantages over other species in sperm RNA functional research on male fertility. The litter size of pigs is the endpoint of a successful developmental process. The present study analysed sperm function and in vivo fertility data to reveal whether the selected genes have practical importance and attempted to develop precise markers to increase field fertility.

High throughput “omics” technologies have provided growing evidence indicating that SIFs, particularly protein and RNA, are involved in the fertilisation process. Numerous proteomic technologies have aided in elucidating the vital role of sperm proteins in developmental biology, and several functional biomarkers have been validated for use in the biomedical field. However, owing to the seminal nature of sperm RNA research, the function of sperm RNA remains a subject of debate. Furthermore, despite the existence of compelling sperm transcriptome studies [50–54], identifying functional biomarkers that can be used in the biomedical field remains a challenge. Functional studies of sperm RNA have mainly focused on small RNAs, including tRNA-derived small RNAs and small interfering RNAs [55–57]. Nonetheless, the function of sperm mRNA in the fertilisation process remains controversial. Pang et al. [39] recently optimised an RT-qPCR-based method to characterise mRNA expression in boar spermatozoa and identified several functional mRNA biomarkers. Interestingly, mRNA markers exhibited a 60–95% overall accuracy in predicting male fertility and significantly increased the litter size by a maximum of 1.61 piglets per insemination in field trials [18, 39, 58]. However, examining only a few functional biomarkers cannot explain the entire fertilisation process. Therefore, it would be desirable to use additional markers to predict male fertility.

Our selected genes were classified using the Gene Ontology database as integral membrane components. Among the genes in this category, *AVPR2*, *ACTB*, *PHB*, and *HSPD1* are considered mRNA markers of male fertility in pigs based on relevant literature. To comprehensively understand the effect of the differential mRNA expression of the selected genes on male fertility, several parameters (sperm motility, motion kinematics, capacitation, and litter size) were analysed against the mRNA expression of the aforementioned genes.

Sperm motility and motion kinematics are important characteristics that can enable sperm to travel through the female reproductive tract to fertilise the oocyte. Additionally, sperm cells must undergo essential maturation changes, such as capacitation and acrosome reaction, to fertilise the oocytes [59]. Therefore, we used sperm motility, motion kinematics, and capacitation status as the pre-fertilisation features of spermatozoa. Although numerous studies have addressed the relationship between conventional semen analyses and male fertility, the exact relationship between these parameters remains not fully understood [32, 60, 61]. In the present study, neither of these pre-fertilisation parameters exhibited a clear correlation with litter size. This was consistent with a previous study [44], which reported that sperm motility, motion kinematics, and capacitation status are better suited for the acquisition of preliminary and quantitative sperm fertility data than of qualitative information.

Transcriptomic biomarkers can explain many biological processes through high-throughput data and are more cost-effective than other biomarkers [62]. Although spermatozoa contain a small quantity of RNA, functional RNAs synthesised during spermatogenesis are transferred to oocytes during fertilisation [63]. The present study suggests that *HSPD1* mRNA plays a functional role in male fertility and could be a biomarker for successful fertilisation.

HSPD1 plays important roles in various biological functions associated with fertilisation. In mouse spermatozoa, tyrosine phosphorylation of the HSPD1 protein was observed, and this protein is involved in receptor-mediated interactions with female gametes [29]. In the present study, the protein level of HSPD1 was checked after the evaluation of *HSPD1* as a sperm mRNA marker. HSPD1 protein was highly expressed in the high-litter size group. High expression of HSPD1 might facilitate receptor-mediated interactions between spermatozoa and oocytes and consequently increase litter size. In the present study, the expression patterns of HSPD1 protein and mRNA were inversely related. On the genomic scale, protein abundance and mRNA expression levels show poor correlation [64]. Moreover, in spermatozoa, neither mRNA functions nor mRNA-protein interactions are known. Evaluation from an evolutionary perspective can help us understand how *HSPD1* mRNA expression is associated with fertilisation [65]. During male gametogenesis in *Arabidopsis*, short suspensor transcripts are generated and transferred to female gametes for zygotic translation. As such, *HSPD1* mRNA might have a separate function in addition to its protein translation, but further research is required.

HSPD1 plays an important role in embryonic development [66]. In the present study, despite the significant correlation between *HSPD1* mRNA expression and sperm motility, motility parameters were not correlated to litter size. This result suggests that *HSPD1* mRNA affects male fertility, irrespective of sperm function. Moreover, the current study suggests that there are sperm variables that impact fertilisation ability or embryo development that are not detectable using standard sperm evaluation methods. Our study demonstrates the relationship between gene expression and the endpoint of the fertilisation process, suggesting that *HSPD1* mRNA expression might be transferred to the oocytes and modulate the entire developmental process, including implantation, foetal development, and successful birth.

## Conclusions

Among the several known sperm membrane protein genes, *HSPD1* mRNA levels were found to be crucial indicators of male fertility. Most importantly, these transcriptomic markers were more closely related to male fertility parameters, especially the litter size of inseminated sows, with *HSPD1* being particularly correlated with sperm motility and motion kinematics. Our findings suggest that *HSPD1* plays a crucial role in fertilisation and developmental processes beyond fertilisation. Therefore, *HSPD1* mRNA could be used in sperm evaluation to predict male fertility before AI which would lead to further improvements in field fertility.

## Supplementary Information

**Additional file 1: Table S1.** Quantitative reverse transcription-polymerase chain reaction (RT-qPCR) primers. **Table S2.** The results for the normality test of every parameters (pre-fertilization parameters, litter size, and gene expression) from 21 boars. **Table S3.** Correlation between pre-fertilisation parameters (sperm motility, motion kinematics, and capacitation status) and gene expression. **Table S4.** Comparison between all parameters in the heat shock protein family D member 1 (*HSPD1*) high and low expression groups. **Fig. S1.** The standard curve and qPCR efficiency of studied genes. **Fig. S2.** The melt curve and gel electrophoresis image of qPCR products. **Fig. S3.** *ACTB, PHB, HSPD1,* and *AVPR2* mRNA expression in high- and low-litter size boar spermatozoa.

## Data Availability

The datasets from the current study are available from the corresponding author upon reasonable request.

## Abbreviations

HSPD1: Heat shock protein family D member 1
SIFs: Sperm intrinsic factors
AVPR2: Arginine vasopressin receptor 2
ACTB: Beta-actin
PHB: Prohibitin
PI3K/AKT: Phosphoinositide 3-kinase/serine-threonine kinase
ROC: Receiver operating characteristic
CASA: Computer-assisted sperm analysis
HYP: Hyperactivated motility
VCL: Curvilinear velocity
VSL: Straight-line velocity
VAP: Average path velocity
ALH: Mean amplitude of lateral head displacement
BCF: Beat cross frequency
LIN: Linearity
WOB: Wobble
PBS: Phosphate-buffered saline
CTC: Chlortetracycline
NaCl: Sodium chloride
RT-qPCR: Quantitative reverse transcription-polymerase chain reaction
GAPDH: Glyceraldehyde 3-phosphate dehydrogenase
SDS-PAGE: Sodium dodecyl sulphate-polyacrylamide gel electrophoresis
HRP: Horseradish peroxidase
SEM: Standard error of the mean
NPV: Negative predictive value
PPV: Positive predictive value
AUC: Area under the curve
