# Supplementary material for: Heat shock protein family D member 1 in boar spermatozoa is strongly related to the litter size of inseminated sows
Source: J Anim Sci Biotechnol. 2022 Apr 15;13:42. doi: 10.1186/s40104-022-00689-0 (PMC9012035; doi:10.1186/s40104-022-00689-0)

**Heat shock protein family D member 1 in boar spermatozoa is strongly related to the litter size of inseminated sows**

Won-Ki Pang, Ji-Hyun Son, Do-Yeal Ryu, Md Saidur Rahman, Yoo-Jin Park, and Myung-Geol Pang*

Department of Animal Science & Technology and BET Research Institute, Chung-Ang University, Anseong, Gyeonggi-do, 17546, Republic of Korea

* Corresponding Author: Tel: +82.31.670.4841; Fax: +82.31.670.3019; Email: mgpang@cau.ac.kr

**Supplementary Tables and Figures**

**Table S1:** Quantitative reverse transcription-polymerase chain reaction (RT-qPCR) primers.

**Table S2:** The results for the normality test of every parameters (pre-fertilization parameters, litter size, and gene expression) from 21 boars.

**Table S3:** Correlation between pre-fertilisation parameters (sperm motility, motion kinematics, and capacitation status) and gene expression.

**Table S4:** Comparison between all parameters in the heat shock protein family D member 1 (HSPD1) high and low expression groups.

**Figure S1:** The standard curve and qPCR efficiency of studied genes.

**Figure S2**: The melt curve and gel electrophoresis image of qPCR products.

**Figure S3**: *ACTB, PHB, HSPD1,* and *AVPR2* mRNA expression in high- and low-litter size boar spermatozoa.

**Table S1:** Quantitative reverse transcription-polymerase chain reaction (RT-qPCR) primers.

| Gene symbol | Upper primer (5'-3') | Tm (℃) | Lower primer (5'-3') | Tm (℃) | Amplicon size (bp) | Reference sequence |
| --- | --- | --- | --- | --- | --- | --- |
| *AVPR2* | CTG CTG TCC ACG GTC TTT GT | 65.3 | AAG TGG CCG ATG AAG ACG TG | 67.4 | 115 | ENSSSCG00000012789 |
| *ACTB* | GAT CTG GCA CCA CAC CTT CT | 64.2 | CTT CTC ACG GTT GGC TTT GG | 66.8 | 102 | ENSSSCG00000007585 |
| *PHB* | ACA TCA CCC TGC GTA TCC TC | 63.9 | GAG GAT CTC TGT GGT GAT GGA | 64.2 | 115 | ENSSSCG00000017549 |
| *HSPD1* | AGA TGG TGT GAC TGT TGC AA | 61.9 | CCC AGC CTC TTC GTT TGT GT | 66.4 | 105 | ENSSSCG00000016077 |
| *GAPDH* | AAG AGC ACG CGA GGA GGA | 66.4 | GGG GTC TGG GAT GGA AAC T | 65.1 | 109 | ENSSSCG00000000694 |
| *PPIA* | TGGCAAAGTGAAAGAGGGCA | 65.5 | TTGTCCACAGTCAGCAATGG | 63.6 | 103 | ENSSSCG00000016737 |

**Table S2:** The results for the normality test of every parameters (pre-fertilization parameters, litter size, and gene expression) from 21 boars.

|  | Shapiro-Wilk | |
| --- | --- | --- |
|  | *P-value* | Data distribution |
| Litter size | 0.08 | Normal distribution |
| MOT, % | 0.10 | Normal distribution |
| HYP, % | 0.02 | Non-normal distribution |
| VCL, ㎛/s | 0.43 | Normal distribution |
| VSL, ㎛/s | 0.00 | Non-normal distribution |
| VAP, ㎛/s | 0.03 | Non-normal distribution |
| LIN, % | 0.08 | Normal distribution |
| BCF, Hz | 0.02 | Non-normal distribution |
| WOB, % | 0.04 | Non-normal distribution |
| ALH, ㎛/s | 0.13 | Normal distribution |
| AR, % | 0.00 | Non-normal distribution |
| F, % | 0.00 | Non-normal distribution |
| B, % | 0.00 | Non-normal distribution |
| TNFRSF19 | 0.11 | Normal distribution |
| HSPD1 | 0.19 | Normal distribution |
| AVTB | 0.23 | Normal distribution |
| PHB | 0.03 | Non-normal distribution |
| AVPR2 | 0.01 | Non-normal distribution |

**Table S3:** Correlation between fertilisation parameters (litter size, sperm motility, motion kinematics, and capacitation status) and gene expression. Left side of table cell is correlation coefficient when gene expression is normalized by *GAPDH*. Right side of table cell is correlation coefficient when gene expression is normalized by *PPIA*. **P* < 0.05.

|  | Litter size | MOT (%) | HYP (%) | VCL (µm/s) | VSL (µm/s) | VAP (µm/s) | LIN (%) | BCF (Hz) | WOB (%) | ALH (µm/s) | AR(%) | F(%) | B(%) | *HSPD1* | *AVPR2* | *ACTB* | *PHB* |
| --- | --- | --- | --- | --- | --- | --- | --- | --- | --- | --- | --- | --- | --- | --- | --- | --- | --- |
| Litter size |  | -0.22 | -0.12 | -0.24 | -0.08 | -0.17 | 0.12 | 0.32 | 0.08 | -0.23 | 0.10 | 0.04 | -0.08 | -0.44*/-0.44* | -0.17/-0.14 | -0.13/0.24 | 0.24/-0.33 |
| MOT |  |  | 0.42 | 0.55* | 0.48* | 0.60* | 0.280 | -0.40 | 0.35 | 0.60* | -0.27 | -0.15 | 0.27 | 0.67*/0.54* | 0.22/0.13 | 0.11/-0.16 | 0.15/0.26 |
| HYP |  |  |  | 0.83* | 0.21 | 0.44* | -0.42 | -0.81* | -0.42 | 0.71* | -0.36 | 0.2 | -0.26 | 0.37/0.20 | -0.35/0.23 | 0.03/-0.08 | 0.54*/0.56* |
| VCL |  |  |  |  | 0.65* | 0.80* | 0.06 | -0.92* | -0.01 | 0.98* | -0.08 | -0.06 | 0.09 | 0.49*/0.31 | 0.03/0.09 | 0.12/0.18 | 0.28/0.09 |
| VSL |  |  |  |  |  | 0.94* | 0.79* | -0.52* | 0.70* | 0.79* | 0.10 | -0.14 | 0.12 | 0.41/0.41* | 0.22/-0.12 | 0.16/0.34 | 0.02/-0.22 |
| VAP |  |  |  |  |  |  | 0.65* | -0.66* | 0.53* | 0.90* | -0.06 | 0.00 | 0.03 | 0.51*/0.48* | 0.12/-0.05 | 0.17/0.22 | 0.09/0.41 |
| LIN |  |  |  |  |  |  |  | 0.07 | 0.98* | 0.27 | 0.19 | -0.23 | 0.17 | 0.19/0.06 | 0.27/-0.22 | 0.10/0.08 | -0.23/-0.09 |
| BCF |  |  |  |  |  |  |  |  | 0.08 | -0.86* | 0.26 | -0.13 | 0.15 | -0.37/-0.21 | 0.19/-0.04 | 0.03/-0.13 | -0.30/-0.18 |
| WOB |  |  |  |  |  |  |  |  |  | 0.20 | 0.23 | -0.26 | 0.30 | 0.22/0.02 | 0.23/-0.20 | 0.12/0.14 | -0.25/-0.18 |
| ALH |  |  |  |  |  |  |  |  |  |  | -0.03 | -0.11 | 0.13 | 0.50*/0.37 | 0.10/0.08 | 0.15/0.19 | 0.22/0.19 |
| AR(%) |  |  |  |  |  |  |  |  |  |  |  | -0.48* | 0.22 | -0.22/-0.28 | 0.36/-0.14 | 0.12/0.01 | -0.20/0.18 |
| F(%) |  |  |  |  |  |  |  |  |  |  |  |  | -0.92* | -0.18/0.06 | -0.22/0.19 | -0.65*/-0.56* | 0.06/-0.08 |
| B(%) |  |  |  |  |  |  |  |  |  |  |  |  |  | 0.28/0.04 | 0.09/-0.15 | 0.67*/0.41 | -0.14/0.05 |
| *HSPD1* |  |  |  |  |  |  |  |  |  |  |  |  |  |  | 0.27/0.20 | 0.35/0.41 | -0.22/0.03 |
| *AVPR2* |  |  |  |  |  |  |  |  |  |  |  |  |  |  |  | 0.09/0.01 | -0.30/-0.11 |
| *ACTB* |  |  |  |  |  |  |  |  |  |  |  |  |  |  |  |  | -0.10/-0.10 |
| *PHB* |  |  |  |  |  |  |  |  |  |  |  |  |  |  |  |  |  |

**Table S4:** Comparison between all parameters in the heat shock protein family D member 1 (HSPD1) high and low expression groups. **P* < 0.05.

|  | Sperm motility and motion kinematics | | | | | | | | | Sperm capacitation | | |
| --- | --- | --- | --- | --- | --- | --- | --- | --- | --- | --- | --- | --- |
|  | MOT* (%) | HYP  (%) | VCL* (µm/s) | VSL* (µm/s) | VAP* (µm/s) | LIN (%) | BCF (Hz) | WOB* (%) | ALH* (µm/s) | AR  (%) | F  (%) | B  (%) |
| High HSPD1 (≥ 3.1798) | 88.40 ± 1.60 | 13.80 ± 2.66 | 146.03 ± 6.06 | 74.63 ± 5.57 | 86.60 ±4.40 | 51.03 ±2.79 | 11.85 ± 0.25 | 57.29 ± 1.84 | 6.58 ± 0.27 | 2.36 ± 1.05 | 85.16 ± 3.45 | 12.48 ± 3.27 |
| Low HSPD1 (< 3.1798) | 80.07 ± 1.04 | 11.10 ± 1.81 | 128.45 ± 5.23 | 55.06 ± 2.17 | 66.02 ± 1.99 | 43.42 ± 2.22 | 12.52 ± 0.42 | 51.79 ± 1.62 | 5.69 ± 0.19 | 2.60 ± 1.00 | 88.73 ± 1.80 | 8.66 ± 1.25 |

|  | Gene expression | | | | | Artificial insemination result |
| --- | --- | --- | --- | --- | --- | --- |
|  | *TNFRSF19* | *HSPD1** | *AVTB* | *PHB* | *AVPR2* | Litter size |
| High HSPD1 (≥ 3.1798) | 0.76 ± 0.03 | 4.47 ± 0.23 | 1.05 ± 0.02 | 0.64 ± 0.02 | 3.90 ± 0.51 | 12.49 ± 0.24 |
| Low HSPD1 (< 3.1798) | 0.86 ± 0.04 | 1.82 ± 0.22 | 1.03 ± 0.02 | 0.67 ± 0.05 | 2.71 ± 0.54 | 12.97 ± 0.23 |

**Figure S1:** The standard curve and qPCR efficiency of studied genes. (A) *AVPR2* standard curve. (B) *ACTB* standard curve. (C) *PHB* standard curve. (D) *HSPD1* standard curve. (E) *GAPDH* standard curve. (F) *PPIA* standard curve.


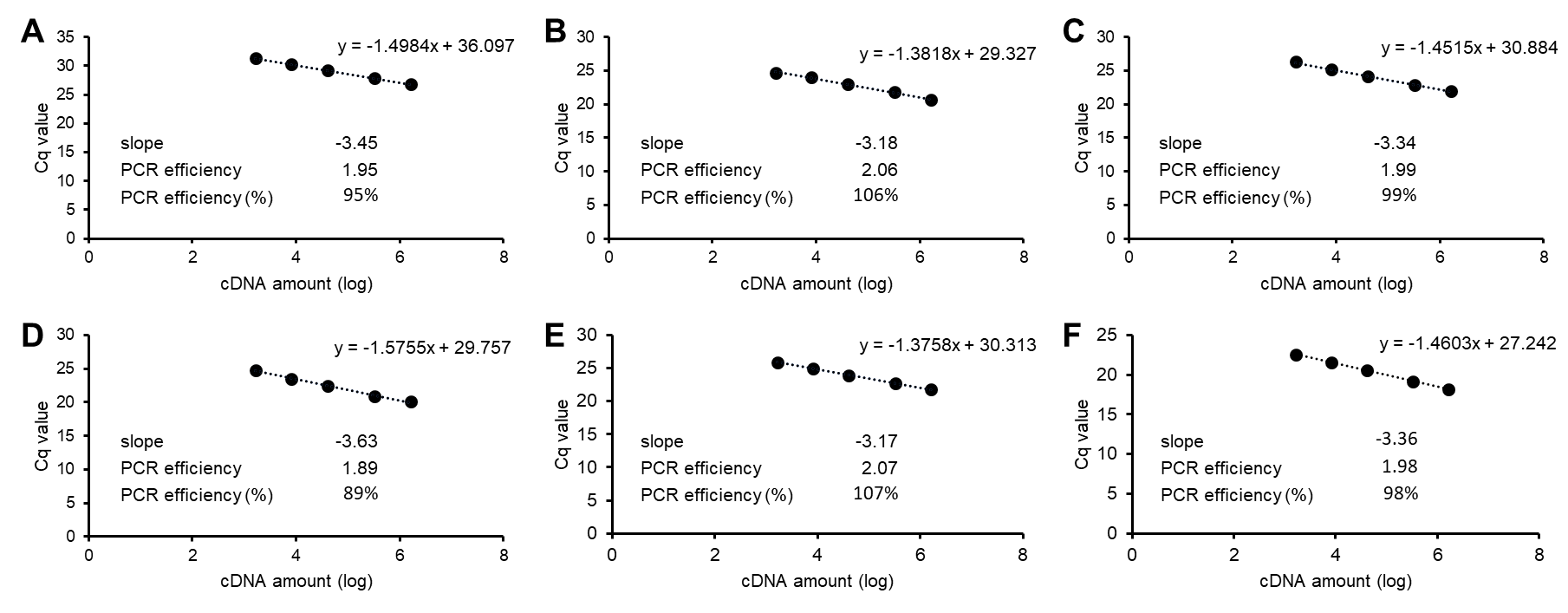


**Figure S2**: The melt curve and gel electrophoresis image of qPCR products. (A) Melting curve analysis of genes. (B) Gel-electrophoresis image of qPCR products.


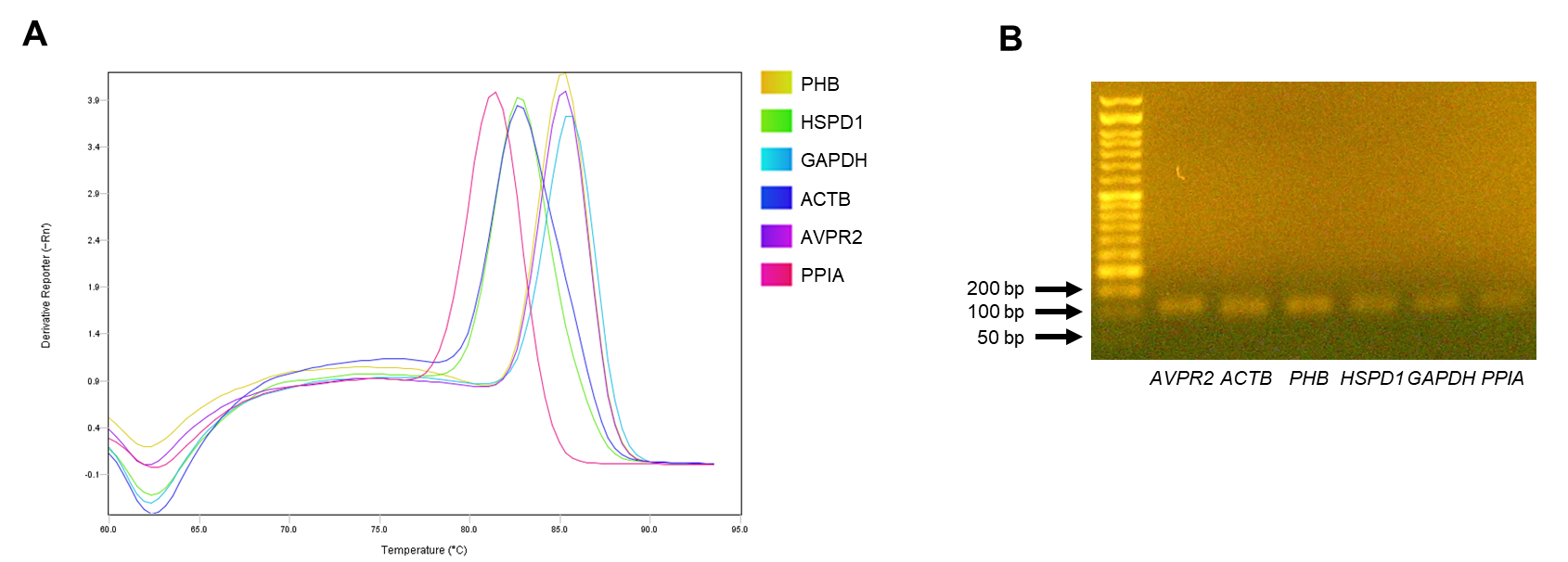


**Figure S3**: *ACTB, PHB, HSPD1,* and *AVPR2* mRNA expression in high- and low-litter size boar spermatozoa.

Differences of marker candidate gene expression in the average litter sizes of the high litter size (*n =* 3) and low litter size (*n =* 3) spermatozoa groups. (A) *AVPR2* mRNA expression in boar spermatozoa with high- and low-litter size. (B) *ACTB* mRNA expression in boar spermatozoa with high and low litter size. (C) *PHB* mRNA expression in boar spermatozoa with high and low litter size. (D) *HSPD1* mRNA expression in boar spermatozoa with high and low litter size. The relative expression was normalized with *PPIA* expression. The data are expressed as the mean ± standard error of the mean (SEM); ^*^*P* < 0.05.


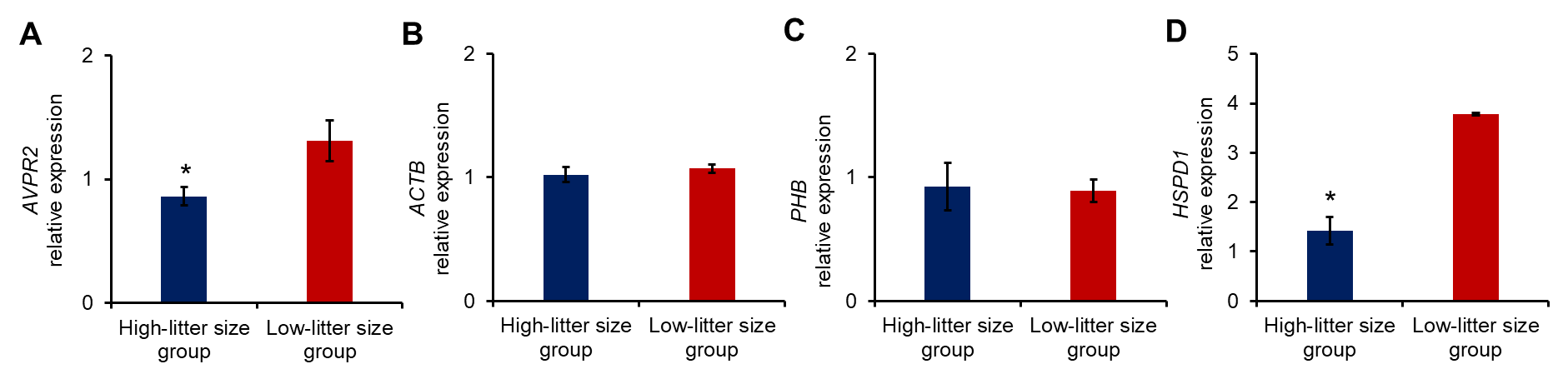

Supplement: Supplementary file 1 — Additional file 1: Table S1. Quantitative reverse transcription-polymerase chain reaction (RT-qPCR) primers. Table S2. The results for the normality test of every parameters (pre-fertilization parameters, litter size, and gene expression) from 21 boars. Table S3. Correlation between pre-fertilisation parameters (sperm motility, motion kinematics, and capacitation status) and gene expression. Table S4. Comparison between all parameters in the heat shock protein family D member 1 (HSPD1) high and low expression groups. Fig. S1. The standard curve and qPCR efficiency of studied genes. Fig. S2. The melt curve and gel electrophoresis image of qPCR products. Fig. S3. ACTB, PHB, HSPD1, and AVPR2 mRNA expression in high- and low-litter size boar spermatozoa. [file 40104_2022_689_MOESM1_ESM.docx]
